# Collateral Supply in Preclinical Cerebral Stroke Models

**DOI:** 10.1007/s12975-021-00969-3

**Published:** 2021-11-19

**Authors:** Philippe Bonnin, Nathalie Kubis, Christiane Charriaut-Marlangue

**Affiliations:** 1 APHP, Physiologie Clinique - Explorations Fonctionnelles, Hôpital Lariboisiere, Université de Paris, 2 rue Ambroise Paré, F-75010 Paris, France; 2 INSERM U1148, LVTS, Hôpital Bichat, Université de Paris, F-75018 Paris, France; 3 INSERM U1141, NeuroDiderot, Hôpital Robert Debré, Université de Paris, 48 bd Sérurrier, F-75019 Paris, France

**Keywords:** Stroke, Preclinical studies, Rat, Mouse, Collateral supply, Circle of Willis, Leptomeningeal anastomosis

## Abstract

Enhancing the collateral blood supply during the acute phase of cerebral ischemia may limit both the extension of the core infarct, by rescuing the penumbra area, and the degree of disability. Many imaging techniques have been applied to rodents in preclinical studies, to evaluate the magnitude of collateral blood flow and the time course of responses during the early phase of ischemic stroke. The collateral supply follows several different routes at the base of the brain (the circle of Willis) and its surface (leptomeningeal or pial arteries), corresponding to the proximal and distal collateral pathways, respectively. In this review, we describe and illustrate the cerebral collateral systems and their modifications following pre-Willis or post-Willis occlusion in rodents. We also review the potential pharmaceutical agents for stimulating the collateral blood supply tested to date. The time taken to establish a collateral blood flow supply through the leptomeningeal anastomoses differs between young and adult animals and between different species and genetic backgrounds. Caution is required when transposing preclinical findings to humans, and clinical trials must be performed to check the added value of pharmacological agents for stimulating the collateral blood supply at appropriate time points. However, collateral recruitment appears to be a rapid, beneficial, endogenous mechanism that can be stimulated shortly after artery occlusion. It should be considered a treatment target for use in addition to recanalization strategies.

## Introduction

The enhancement of collateral blood supply during the acute phase of cerebral ischemia could limit the extension of the core infarct by rescuing the penumbra area, and the degree of disability. Many studies have tried to image this collateral blood flow (BF) [1–5], and to propose therapeutic strategies for improving the salvage of cerebral penumbral tissue around the core infarct and limiting its extension in stroke patients [6–8].

Many imaging techniques have been applied to rodents in preclinical trials, to evaluate the magnitude of the collateral BF and the timing of responses during the early phase of ischemic stroke. Non-invasive techniques, such as 2D color-coded Doppler imaging, can be used [9]. More invasive techniques have also been used, but these require scalp incision, and, in adult rodents, a reduction of skull thickness (single-point laser Doppler flowmetry (LDF); laser speckle contrast imaging (LSCI) [10, 11]) or the creation of a cranial window (Sidestream Dark Field (SDF) [12]; two-photon imaging [13]; and optical coherence tomography (OCT) [14]). These invasive techniques can be used to visualize and quantify collateral recruitment at the surface of the brain, but with a depth of exploration limited to several microns. Non-invasive ultrasound techniques can be used to monitor BF and to estimate collateral supply over whole hemispheres and in the various arterial territories of the brain, with the possibility of sequences of measurements repeated over several days. However, all of these techniques are underused due to their expense and the expertise required for their implementation.

The collateral blood supply follows different routes in the base (the circle of Willis) and surface of the brain (leptomeningeal also called pial arteries), corresponding to the primary and secondary collateral pathways, respectively. In this review, we will first describe the cerebral collateral systems, and will then focus on their modifications following a pre-Willis or post-Willis occlusion in preclinical animal models, before considering the molecular pathways investigated to date. We will then consider the pharmacological agents for stimulating collateral recruitment identified to date, before finally suggesting possible avenues of preclinical research to provide a comprehensive understanding of brain collateral recruitment.

## The Cerebral Collateral Systems

The cerebral arterial supply of rodents, like that of humans, is ensured by four arteries: two internal carotid arteries (ICA) positioned anteriorly and two vertebral arteries positioned posteriorly and feeding into the basilar trunk (BT). However, the rodent system has a particular organization, with each ICA supplying the supratentorial region and giving rise to the three cerebral arteries involved in supplying blood to each hemisphere. Each ICA gives rise to two side branches — the posterior cerebral artery (PCA) and the middle cerebral artery (MCA) — and one terminal artery, the anterior cerebral artery (ACA). The BT essentially supplies the infratentorial region, the cerebellum and the brainstem. The cerebral arterial circulation includes two native pre-existing arterial collateral systems. The proximal system, known as the circle of Willis, is interposed between the two ICAs and the BT upstream, and the cerebral arteries downstream. The distal system is the native pre-existing end-to-end leptomeningeal anastomoses that develop at the surface of the cortex of each hemisphere, distally joining the three cerebral arterial networks on each side.

### The Circle of Willis in Rodents

**Fig. 1:**
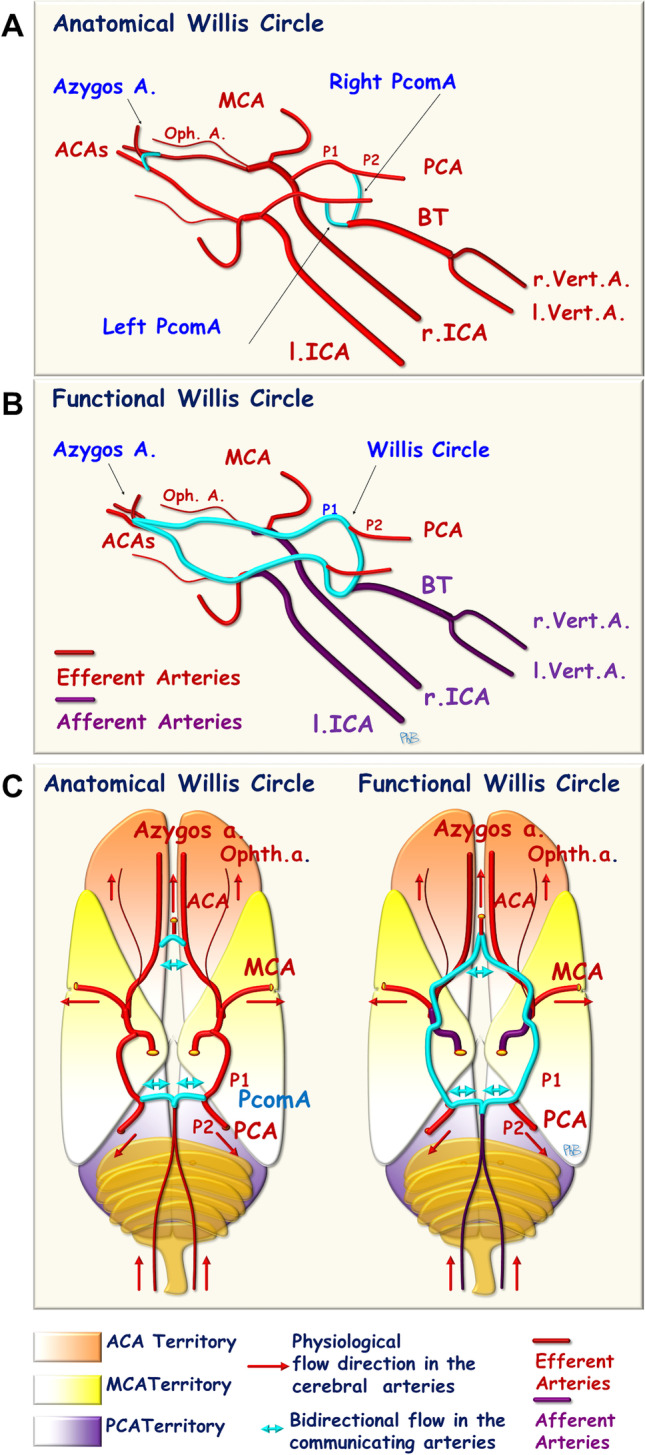
The circle of Willis (left posterolateral view). **A** Anatomical representation. **B** Functional representation (purple: “afferent” arteries, red: “efferent” arteries). **C** Horizontal representation of the circle of Willis. ACA, anterior cerebral artery; PCA, posterior cerebral artery; ICA, internal carotid artery; BT, basilar trunk; Oph A, ophthalmic artery; PcomA, posterior communicating artery; vert. A, vertebral artery; r, right; l, left

In rodents, the two ACAs join at the front to give rise to the azygos artery, the equivalent of the anterior communicating artery in humans. The BT gives rise to one posterior communicating artery (PcomA) on each side, extending to the corresponding PCA. The circle of Willis consists of the following structures, caudal to rostral: the BT, the PcomA, the P1 segment of the PCA, the end of the ICA, and the ACA on each side joining the azygos artery (Fig. 1A) [15]. Doppler ultrasound imaging has shown that flow in the PcomAs is low and bidirectional due to the small difference in arterial pressure between the ICAs and the BT. In the P1 segment of the PCAs, flow normally occurs in a forward direction, from the ICA to the P2 segment of the PCA.

### Cortical Leptomeningeal Anastomoses

**Fig. 2:**
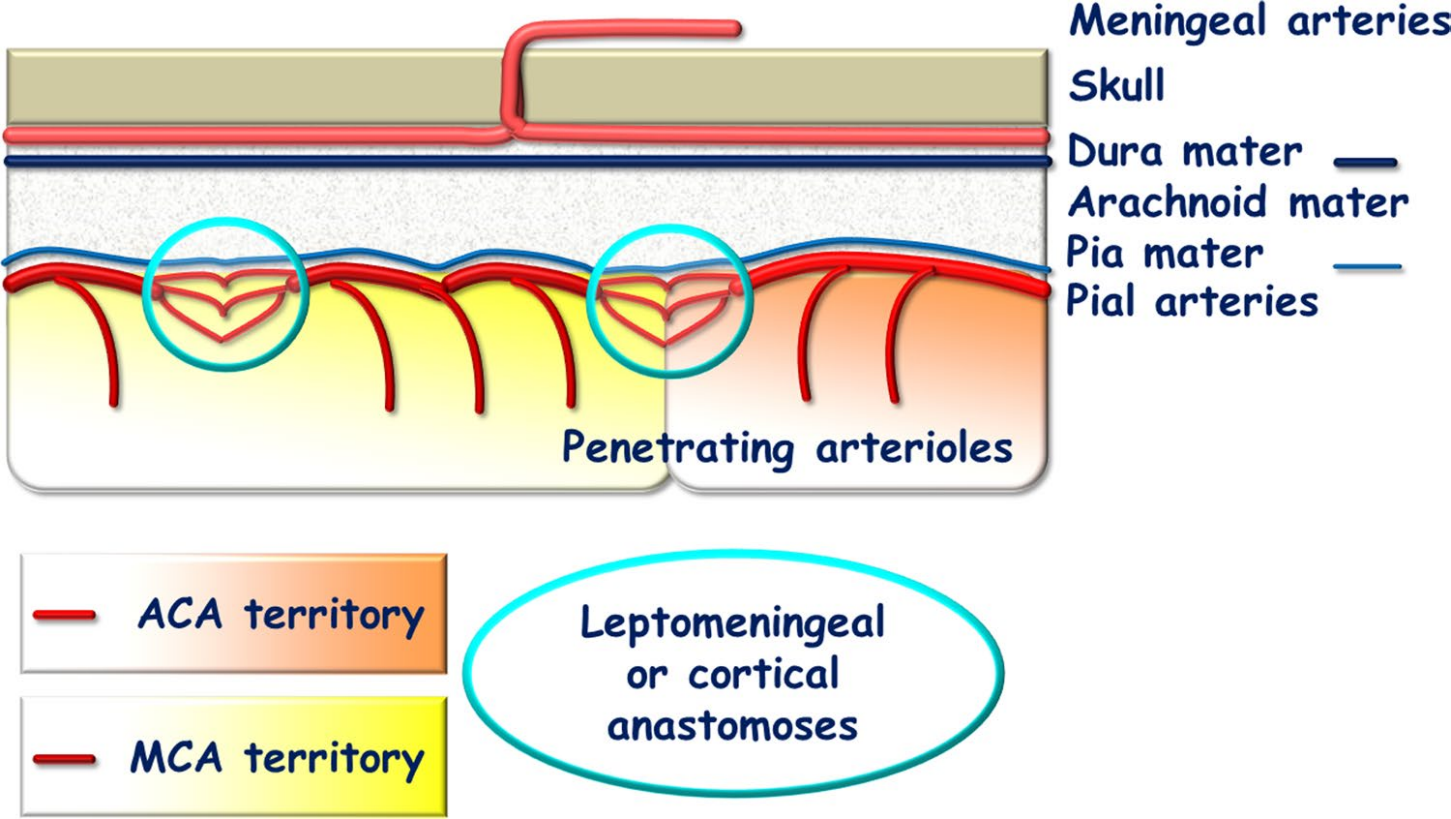
Schematic representation of the leptomeningeal artery anastomoses

**Fig. 3:**
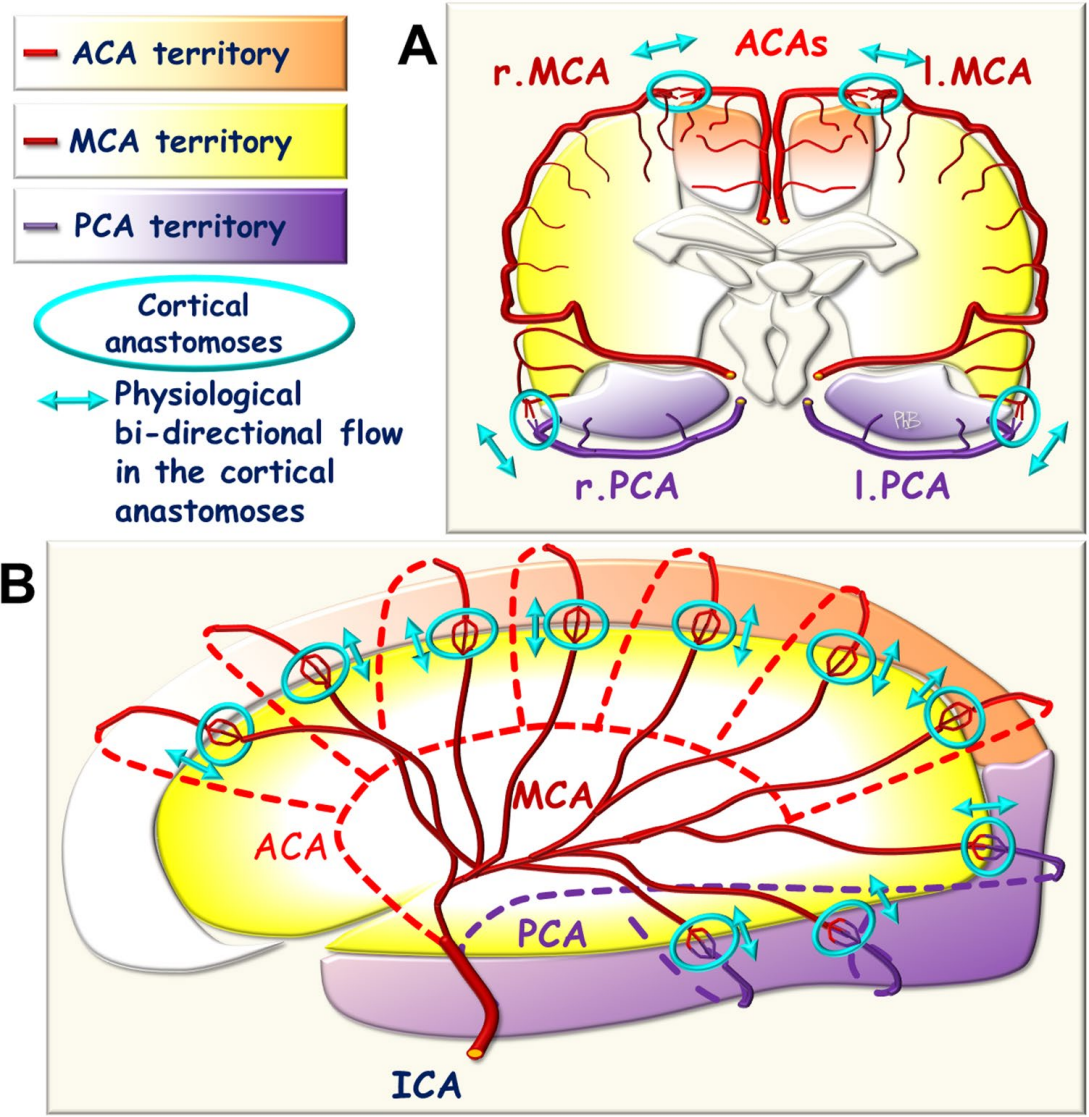
Schematic representation of **A** coronal and **B** sagittal views of the leptomeningeal, cortical anastomoses extending between the different arterial cerebral territories

There are cortical anastomoses extending between the terminal branches of each cerebral artery at the surface of the cortex and into the leptomeningeal space, just before the pial arteries penetrate into the gray mater (penetrating arteries) (Figs. 2 and 3). The presence of these numerous anastomoses at the edge of each arterial cerebral territory in each hemisphere transforms each cerebral arterial network into a non-terminal vascular network with the conservation of collateral BF in cases of cerebral artery or branch occlusion. In normal conditions, arterial blood pressure is similar on either side of these anastomoses, so there is no pressure gradient and BF is low, intermittent, and bidirectional, following normal slight pressure fluctuations.

As in humans, a collateral supply system may be established between the external carotid artery and the ICA in cases of ICA proximal occlusion, but this vascular collateral supply system has not yet been fully studied in rodents.

## Collateral Systems in Cases of Arterial Occlusion

### Occlusion of a Pre-Willis Artery

**Fig. 4:**
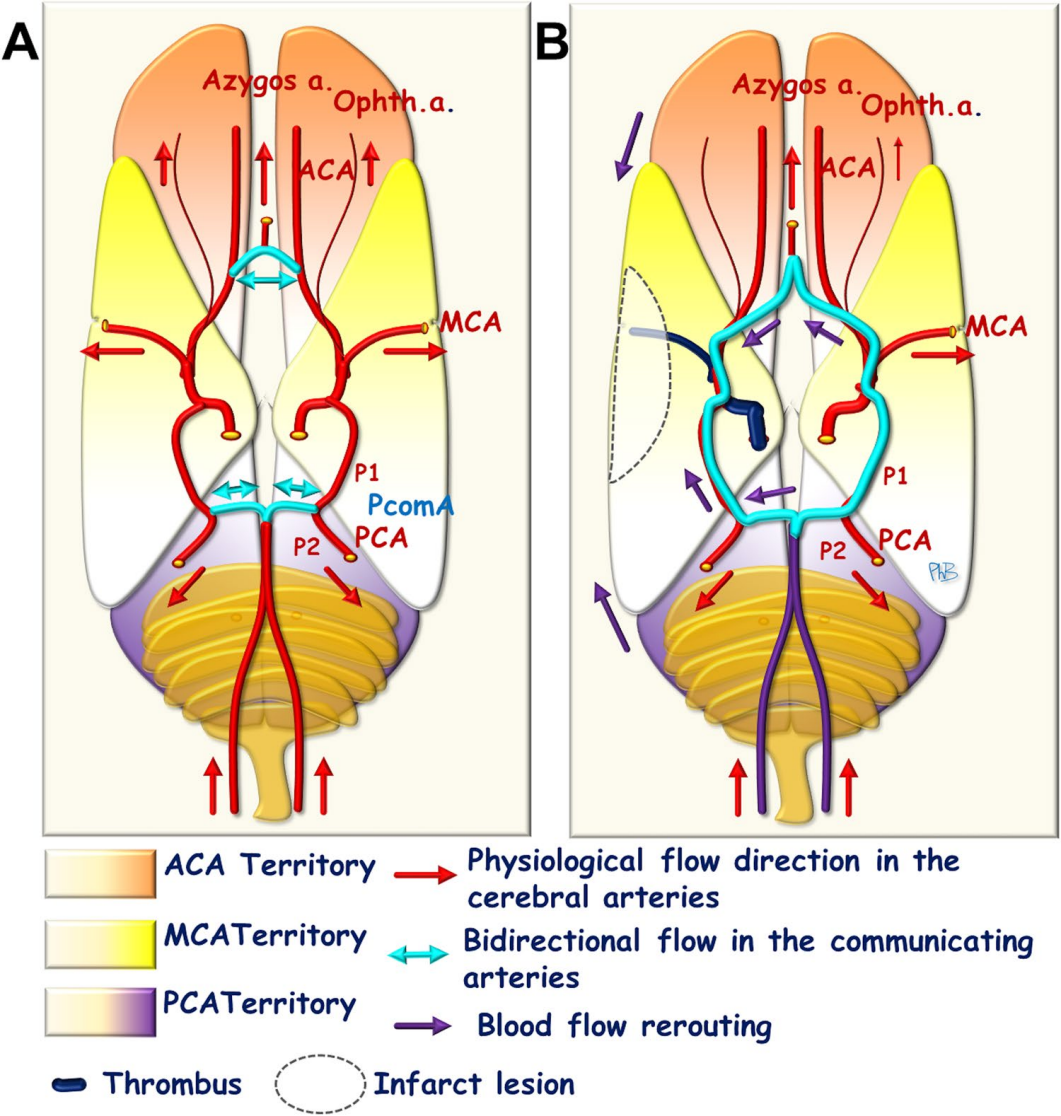
Schematic representation of the blood flow towards the circle of Willis. **A** Physiological distribution. **B** Rerouting of blood flow in the case of a pre-Willis arterial occlusion (ICA)

If one of the ICAs is occluded, then the circle of Willis and the pre-existing cortical (leptomeningeal) anastomoses are used to establish a collateral supply to counteract BF deprivation and the ensuing ischemic effects in the ipsilateral hemisphere. The occlusion of the artery triggers a decrease in arterial blood pressure, to almost zero, in all ipsilateral cerebral arteries, and a decrease in hemodynamic resistance in the corresponding cerebral territories, following microvascular dilation in response to local hypoxia–ischemia. The creation of a pressure gradient directs BF into the various segments of the circle of Willis, towards the cerebral arteries on the side of the carotid occlusion, and through the cortical anastomoses. The magnitude of the decreases in arterial pressure and hemodynamic resistance control the levels of collateral blood supply though the different anastomotic systems. The internal diameters of the anastomotic vessels also control the efficacy of the collateral supply and BF rerouting into the two collateral systems. In the rostral segment of the circle of Willis, arterial pressure remains normal on the contralateral side, but falls on the ipsilateral side. BF follows the pressure gradient. It therefore increases in the contralateral ICA and the contralateral proximal ACA, to ensure (1) the anterograde perfusion of the azygos artery, and (2) the retrograde perfusion of the ipsilateral proximal ACA, towards the ipsilateral MCA. In the caudal part of the circle of Willis, unidirectional flow is established in the ipsilateral PcomA, from the BT towards the PCA, to supply (1) the distal PCA and (2) the proximal PCA, with a reversal of flow towards the ICA and the MCA (Fig. 4). Arterial blood pressure is reasonably well preserved in the azygos artery, and even in the distal PCA. A pressure gradient is, therefore, established on either side of the numerous cortical anastomoses between the ipsilateral ACA or PCA territories and the MCA territory. This leads to the establishment of a cortical collateral supply system carrying blood towards the MCA territory. In cases of permanent common carotid artery (CCA) occlusion, flow reversal can occur in the ipsilateral ICA for a few days, directing blood towards the carotid bifurcation to supply the external carotid artery (ECA) [16].

### Occlusion of a Post-Willis Artery

**Fig. 5:**
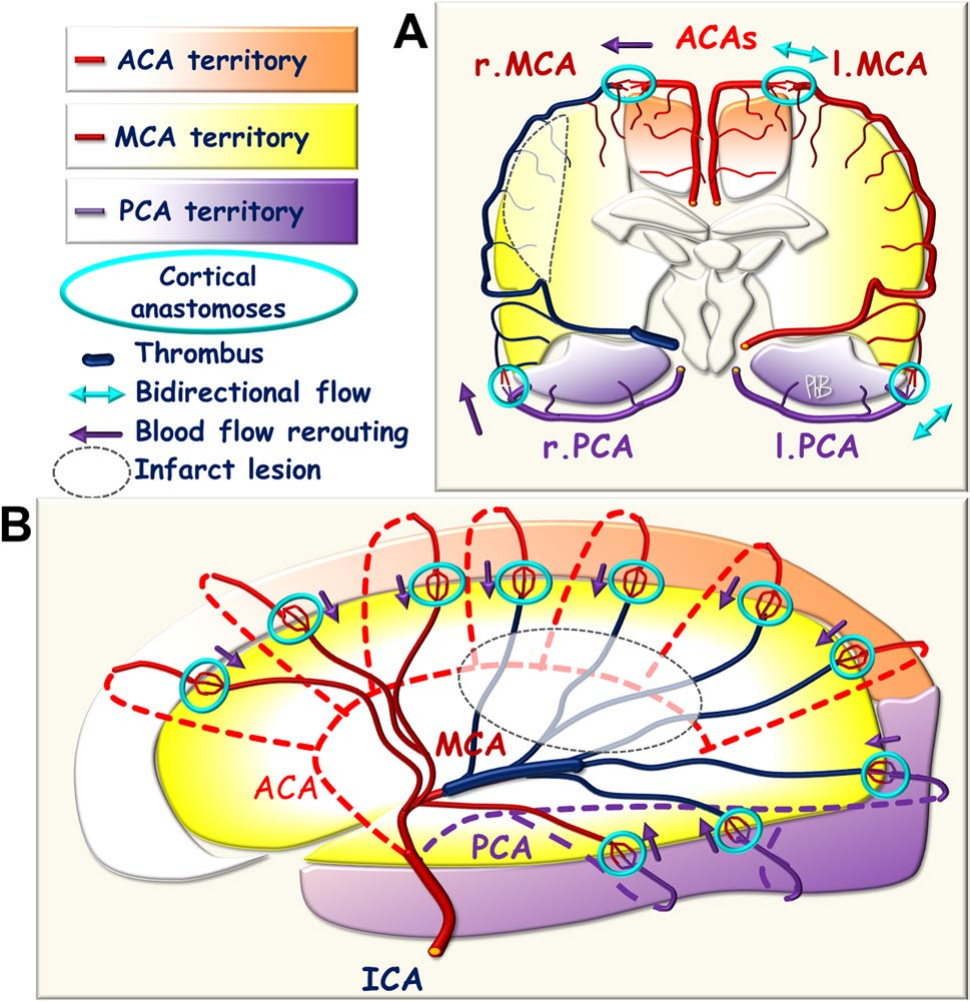
Schematic representation of the blood flow towards the leptomeningeal anastomoses in the case of post-Willis arterial occlusion (ICA). **A** Coronal view. **B** Sagittal view

In cases of occlusion of one of the three cerebral arteries, the consecutive decrease in arterial blood pressure and arterial resistance is limited and focused on a single cerebral arterial territory. A pressure gradient results, and is exerted on either side of the cortical anastomoses extending between the neighboring cerebral territories, triggering unidirectional BF into the anastomoses in the so-called watershed zone. For instance, in the case of an MCA occlusion, collateral blood supply to the leptomeningeal arteries is established, extending between the ACA and MCA and between the PCA and MCA territories towards the MCA territory, in which blood pressure and hemodynamic resistance have fallen. BF must therefore increase upstream, in the ACA and PCA, with the corresponding upstream modifications in the circle of Willis (Fig. 5).

A functional interpretation can therefore be applied to the circle of Willis. This structure receives afferent arteries (the two ICAs and the BT), and the three cerebral arteries on each side can be considered efferent arteries of the circle of Willis (Fig. 1B). Anatomical variations of the circle of Willis therefore also determine the final brain outcome. In rodents, anatomical variation of the azygos artery, equivalent to the anterior communicating artery in humans, is extremely rare and does not appear to limit the establishment of a collateral blood supply via the anterior part of the circle of Willis. However, bilateral or unilateral PcomA patency plays a role in limiting the establishment of collateral blood supply via the posterior part of the circle of Willis. Differences have been observed between strains of mice. Moreover, the remodeling of PcomA induced by age and hypertension may decrease PcomA diameter, and PcomA flow-mediated outward remodeling after unilateral permanent MCA occlusion has been reported to be dependent on genetic background and greater on the ipsilateral side [17–21]. In humans, circle of Willis variations are more common. In a series of stroke and control patients undergoing vascular imaging (magnetic resonance angiography, computed tomography angiography (CTA), and/or digital subtraction angiography (DSA)), up to 62% of patients presented vascular variants, with 22% having variants of both the anterior and posterior circulation. The most prevalent vascular variant was agenesis/hypoplasia of the ACA (18%), followed by the unilateral fetal type of posterior cerebral artery (12%) [22]. In a series of 376 stroke patients, the group with the poorest prognosis had incomplete anterior and/or posterior segment of the circle of Willis [23]. Collateral blood supply via the cortical anastomoses must be established effectively and rapidly, to prevent lesion extension; the efficacy and rapidity of this process are determined by the degree of vasodilation, which has been shown to depend on the nitric oxide (NO) pathway allowing proportional sustained collateral BF [24]. In cases of endothelial dysfunction, the collateral supply is insufficient to counteract ischemia in the brain tissues, and results in larger brain lesions [25].

## Preclinical Stroke Models and Collateral Supply Efficacy

The efficacy of collateral recruitment has been investigated in preclinical models. It depends on the species (rat or mouse), rodent strain, and experimental model (Table 1).

**Table 1:** Collateral supply in preclinical stroke studies in control conditions (no treatment) according to the developmental stage in the last 10 years

Animal	Strain	Age	Ischemic procedure	Collateral* supply from	Imaging technique	Outcomes		References
						Cellular effects	Brain lesion effects	
Mouse	C57BL/6	P90	Occlusion of the pial arteriole	Leptomeningeal anastomoses (180 min)	Two-photon	Cell death delayed	Lesion: decrease	[26]
Mouse	C57BL/6	P90	pMCAo + tCCAo	Leptomeningeal anastomoses (1 and 24 h)	Optical coherence tomography	-	Lesion: decrease Better functional score	[14]
Mouse	Balb/C	P90	pMCAo + tCCAo	Leptomeningeal anastomoses (1 and 24 h)	Optical coherence tomography	-	Lesion: + + Worse functional score	[14]
Mouse	C57BL/6	P70	BCCAs	Circle of Willis (PcomA) and leptomeningeal anastomoses (24 h)	MRA and arterial spin labeling CBF	PcomA patency and collateral vessels influence the lesion size		[27]
Mouse	C57BL/6	P70	tMCAo	Circle of Willis (PcomA) and Leptomeningeal anastomoses (24 h)	MRA and arterial spin labeling CBF	PcomA patency and collateral vessels influence the lesion size		[27]
Rat pup	Wistar	P7	Left pMCAo + left tCCAo	Leptomeningeal anastomoses (ACA & PCA to MCA—1 h)	2D color-coded US imaging	-	48% with no lesion	[9]
Rat pup	Wistar	P7	left pMCAo + left tCCAo	Leptomeningeal anastomoses (ACA & PCA to MCA - 1 h)	2D-color coded US imaging	Correlation between mBFVel in the PCA and lesion score		[9]
Rat pup	Wistar	P7	Left pMCAo + left and right tCCAo	Leptomeningeal anastomoses (ACA and PCA to MCA—1 h)	2D color-coded US imaging	-	24% with no lesion	[9]
Rat pup	Wistar	P7	left pMCAo + left & right tCCAo	Leptomeningeal anastomoses (ACA & PCA to MCA -1 h)	2D-color coded US imaging	Correlation between mBFVel in the BT and lesion score		[9]
Dog	Mongrel	Adult	pMCAo (M1)	Pial collaterals	DSA	Mathematical modeling between lesion growth and pial collateral assessment		[28]

*The timing indicates the time at which measurements have been done. *P9*, 9-day-old; *BCCAS*, bilateral common carotid artery stenosis; *pMCAo*, permanent MCA occlusion; *tMCAo*, transient MCAo; *tCCAo*, transient CCA occlusion; *M1*, M1 segment; *ACA*, anterior cerebral artery; *PcomA*, posterior communicant artery; *PCA*, posterior cerebral artery; *mBFVel*, mean blood flow velocity; *US imaging*, ultrasound imaging; *MRA*, magnetic resonance angiography; *CTP*, computed tomography perfusion; *DSA*, digital subtraction angiography.

In the adult mouse brain, two-photon imaging has shown that the occlusion of a pial arteriole leads to an increase in the diameter of its pre-existing leptomeningeal anastomoses, decreasing cell death rates in the penumbra in C57BL/6 mice [26]. Optical coherence tomography and angiography studies of the combination of a permanent MCA occlusion (pMCAo) and transient CCA occlusion (tCCAo) in two different mouse strains showed that native leptomeningeal collateralization accounted for rapid (30 min) retrograde flow recruitment, which continued to improve over 7 days in C57BL/6 mice, but not in Balb/C mice [14]. Magnetic resonance imaging (MRI) and magnetic resonance angiography (MRA) have identified three sources for the rerouting of BF in C57BL/6 mice after the induction of a focal stroke. Two of these three sources — the contralateral arterial network and the vertebrobasilar circulation — are essential. The importance of the third source is more debatable, particularly during the acute phase of stroke; it involves the creation of retrograde flow from ECA branches towards the ICA. For some authors, the PcomAs could be the main determinant of stroke lesion size, at least during the first week of recovery after stroke induction [27]. As an illustration of the importance of collaterals and the capacity to respond to hemodynamic modifications, it has been shown that genetic selection in rodents native to high elevations has resulted in the presence of abundant leptomeningeal arterial collaterals, to optimize oxygen delivery and to meet oxygen demand in conditions of oxygen limitation. These rodents display almost complete protection against stroke, much stronger than that observed in lowland rodents [29]. No clinical reports of a correlation between stroke severity, elevation, and collateral blood supply have been published. In two neonatal Wistar rat stroke models, left pMCAo and ipsilateral tCCAo resulted in a stroke lesion in only 48% of animals, whereas left pMCAo combined with tCCAo in both CCAs resulted in a stroke lesion in 64% of animals. We used 2D color-coded ultrasound imaging to investigate the hemodynamic mechanisms preventing infarct development in neonatal rats. We showed that, at the end of arterial occlusion (50 min after occlusion, 10 min before left tCCAo release), mean BF velocities (mBFVel) were significantly higher in the right ICA and the BT, with a reversal of BF in the P1 segment of left PCA coming from the PcomA towards the ICA [9]. These observations suggest that there are many anastomoses, with a high degree of patency, in the neonatal rat brain, because no infarcts were observed despite multiple arterial occlusions. In another species, the mongrel dog, MRA showed that pial collateral supply did not develop until 15 min after pMCAo; it was inversely correlated with infarct growth rate index and was even predictive of asymptotic infarct growth [28]. This response, with a very short onset time, is due to flow-mediated vasodilation, as explained above.

## Therapeutic Strategies for Modifying Collateral Supply in Preclinical Stroke Models

Numerous studies have evaluated the impact of treatments on collaterals with different readouts: number of collaterals, tissue damage volume, and cellular and neurological score outcomes. However, despite the availability of several methods for assessing collateral supply during the acute phase of ischemic stroke, these methods are generally underutilized.

### Neonatal and Juvenile Stroke

Only a few studies have used 2D color-coded ultrasound imaging to investigate the effect of treatment on BF rerouting into the circle of Willis and cortical collateral recruitment in the developing brain (Table 2, upper part).

**Table 2:** Collateral supply in preclinical stroke studies after treatment according to the developmental stage, experimental procedure, and rodent strains in the last 10 years

Animal	Strain	Age	Ischemic procedure	Pharmacological agent	Collateral* supply from	Outcomes		References
						Cellular effects	Brain lesion effects	
Mouse pup	C57BL/6	P9	Left pMCAo	Sildenafil®	No increase (90 min)	Microglia M2 + +	Lesion: decrease	[30]
Mouse pup	C57BL/6	P9	Left pMCAo	PJ34 (PARP inhibitor)	Leptomeningeal anastomoses (ACA to MCA) (3 h)	BBB leakage and astrocytes: decrease in rostral	Tissue loss: decrease in rostral	[31]
Rat pup	Wistar	P7	Left pMCAo + left tCCAo	iNO during ischemia	Leptomeningeal anastomoses (PCA to MCA) (End of ischemia)	Cell death, oxidative stress, and microglia decrease	Lesion: decrease	[32]
Rat pup	Wistar	P7	Left pMCAo + left and right tCCAo	Alprostadil® (PgE1)	Leptomeningeal anastomoses (ACA to MCA) (1 h)	GFAP decrease	Lesion: decrease	[33]
Rat juvenile	Wistar	P15	Left pMCAo + left and right tCCAo	7-NI	Leptomeningeal anastomoses (PCA to MCA) (5 to 15 min)	US, LSCI, and SDF imaging Capillaries recruitment and BF index increase		[34]
Rat juvenile	Wistar	P15	left pMCAo + left & right tCCAo	CCAo release: L/R	Leptomeningeal anastomoses (ACA to MCA) (PCA to MCA)	Apoptosis + + Microglia + +	-	[35]
Rat juvenile	Wistar	P15	left pMCAo + left & right tCCAo	CCAo release: R/L	Circle of Willis + Leptomeningeal anastomoses (ACA to MCA) (PCA to MCA)	Apoptosis + / − Microglia +	-	[35]
Mouse	C57BL/6	P90	Left pMCAo	CYM-5442/RP-001 (S1P1 agonists)	Leptomeningeal anastomoses (ACA to MCA + PCA to MCA)	-	Lesion: decrease	[36]
Mouse	C57BL/6	P90	Left tMCAo	CYM-5442 / RP-001 (S1P1 agonists)	Leptomeningeal anastomoses (ACA to MCA + PCA to MCA)	-	Lesion: decrease	[36]
Mouse	C57BL/6	P90	tCCAo	S1PR1 antagonist SEW2871	Leptomeningeal anastomoses (14 days)	Endothelial cells proliferation	Diameter of collateral vessels increase	[37]
Mouse	C57BL/6	P90	pMCAo	S1PR1 antagonist SEW2871	Leptomeningeal anastomoses (14 days)	-	Lesion decrease Functional recovery	[37]
Mouse	C57BL/6	P90	tMCAo	RiPreC	Leptomeningeal anastomoses (45 min)	-	Lesion: decrease	[38]
Mouse	C57BL/6	P90	pMCAo + tCCAo	RiPostC	Leptomeningeal anastomoses (24 h)	Monocytes/macrophages increase	Lesion: decrease	[39]
Mouse	C57BL/6	P90	pMCAo	Sensory stimulation	Leptomeningeal anastomoses (24 h)	No pial collateral supply	No effect on lesion	[40]
Mouse	CD1	P90		Sensory stimulation	Leptomeningeal anastomoses (24 h)	No pial collateral supply	No effect on lesion	[40]
Mouse	C57BL/6	P360	pMCAo	Aerobic exercise	Pial collateral arterioles (3 days)	Increase eNOS and SOD in vascular wall Inflammation reduced	Lesion: decrease Protection from collateral rarefaction	[41]
Mouse	CD1	P60 P80	pMCAo	Therapeutic targeting of EphA4 and/or Tie2	Leptomeningeal and cortical anastomoses Remodeling (3, 7, 14 days)	EphA4 and/or Tie2 receptors	Lesion decrease	[42]
Rat	Wistar	Adult	tMCAo	PHE, PLG, ACZ	Leptomeningeal anastomoses (ACA to MCA) (90 min)	-	Cortical lesion: decrease – functional recovery	[43]
Rat	Wistar	Adult	tMCAo	HDT	Leptomeningeal anastomoses (ACA to MCA) (90 min)	-	Lesion: decrease + + Functional recovery	[41]
Rat	Sprague–Dawley	Aged	pMCAo (M1)	RiPreC	Pial collaterals (2 h)	-	Lesion decrease	[13]
Rat	Sprague–Dawley	Adult	pMCAo	PostC (TAo)	Leptomeningeal and cortical anastomoses (ACA to MCA) (45 min)	LSCI maps of blood flow – vessel diameter increase		[44]
Rat	Wistar (SHRSP)	Adult	pMCAo	Glucose	Leptomeningeal anastomoses (3.5 h)	LSCI—collateral decreased		[45]

*The timing indicates the time at which measurements have been done. *P9*, 9-day-old; *pMCAo*, permanent MCA occlusion; *tMCAo*, transient MCAo; *PJ34*, poly(ADP-ribose) polymerase; *S1P1*, sphingosine 1-phosphate receptor-1; *PgE1*, prostaglandin E1; *PHE*, phenylephrine; *PLG*, polygeline; *ACZ*, acetazolamide; *HDT*, head down tilt; *RiPerC*, remote ischemic per-conditioning; *RiPostC*, remote ischemic postconditioning; *L/R*, left CCAo release 5 min before right CCAo release; *R/L*, right CCAo release 5 min before left CCAo release; *TAo*, transient aortic occlusion; *ACA*, anterior cerebral artery; *BT*, basilar trunk; *MCA*, middle cerebral artery; *PCA*, posterior cerebral artery; *BBB*, blood–brain barrier; *LSCI*, laser speckle contrast imaging; *SDF imaging*, Sidestream Dark Field imaging; *US imaging*, ultrasound imaging; *eNOS*, endothelial NO synthase; *SOD*, superoxide dismutase; *SHRSP*, spontaneous hypertensive stroke-prone rat; *7-NI*, 7 nitro-indazole; *mBFVel*, mean blood flow velocities.

In the brains of neonatal (P9) mice subjected to single pMCAo, sildenafil, a phosphodiesterase-5 inhibitor that prolongs cGMP action in multiple vascular territories, was unable to produce a significant increase BF supply during the first 60 min after pMCAo, but did reduce cell death, inflammation, and lesion size measured 48 h later [30]. In a study using the same ischemic procedure in P9 mice, PJ34, a PARP inhibitor stimulating the endothelial NO synthase, increased BF in the left and right ICA 3 h after occlusion, resulting in smaller lesions, but only in the rostral part [31].

These experiments in neonatal (P9) mice suggest that collateral BF is first established between the ACA and MCA, with several additional hours required to enhance collateral BF between the PCA and MCA. The anastomotic vessels established between the ACA and MCA are greater in both number and diameter than those established between the PCA and MCA [46], potentially accounting for the observation that pMCAo leads to collateral BF preferentially from the rostral part of the brain in mice.

Conversely, in the brains of P7 rats subjected to combined left pMCAo and left (L) and right (R) tCCAo (50 min), a model known to generate stroke lesions in the MCA territory, inhaled NO (iNO), increases mBFVel in the BT at the end of the transient occlusion procedure, this increase being correlated with a decrease in lesion size 48 h later [32]. Moreover, we demonstrated in this model that infarct volumes increased after pan NOS inhibition and/or the inactivation of eNOS alone, leading to nNOS hyperphosphorylation [24, 47]. These conditions result in enhanced oxidative stress and collateral failure [47]. By contrast, in the same model, Alprostadil® (PgE1, 20 µg/kg administered i.p., with 4 injections every 5 min) enhanced collateral supply 1 h after reperfusion for the left ICA (supplying the left hemisphere) only. This collateral supply is unlikely to correspond to a steal phenomenon, although the necessary measurements were not made to confirm this, as PgE1 treatment also reduced thromboxane A-synthase-1 gene expression 2 h after treatment, and reactive astrocyte density and lesion volume after 48 h of recovery [33]. Ultrasound measurements were taken at only one time point (1 h after CCAo release). We therefore cannot exclude the possibility that BF between the MCA and PCA territories (via the BT) increased a few hours later, accounting for the decrease in infarct volume.

In the brains of P15 rats subjected to left pMCAo and L and R tCCAo (60 min), neuronal NO synthase inhibition by 7-NI (25 mg/kg i.p. administered 30 min before ischemia and/or at re-flow) enhances BF in the BT (measured by US imaging), and gradually increases BF (LSC imaging) after 1 to 15 min of re-flow [34]. SDF imaging showed similar increases in the diameter of the MCA branches and the number of cortical capillaries at 5 and 15 min. Total vessel density was found to have increased at 5 min, remaining high at 15 min, and MFI (mean flow index scored from 0 (no flow) to 3 (normal flow)) had increased at 15 min [48]. This paradoxical effect may be explained by the reported opposite effects of different doses of neuronal NO synthase inhibitors [24, 49]. Indeed, 7-NI can induce vasoactive mediators, including endothelial NOS-derived NO, and other molecules, such as prostanoids, acetylcholine, dopamine, histamine, and vasoactive intestinal peptide [50]. We showed that, in juvenile rats treated with 7-NI, the brain responded to ischemia by producing prostaglandins in a COX-2-dependent manner [48]. To our knowledge, no similar evaluation of blood flow indices has been performed in other stroke models.

We also showed, in this model, that unclamping right CCAo 5 min before left CCAo resulted in less brain damage than unclamping left CCAo then right CCAo (a postconditioning procedure), by reducing cell death, inflammation, and reactive nitrogen species levels [35].

### Adult Stroke

Most studies on adult mouse and rat brains (Table 2, lower part) have evaluated the recruitment of leptomeningeal and/or pial and cortical anastomoses through pre- or postconditioning treatment with pharmacological drugs, leading to a smaller infarct volume and better functional recovery. In adult rats, the establishment of a collateral blood supply has been observed 60 min after tMCAo and the application of various therapeutic strategies (phenylephrine, polygeline, acetazolamide, and/or head down tilt) [43]. The increase in microvascular perfusion was measured by SDF imaging 2–2.5 h after pMCAo in S1p1^*ECKO*^ mice [36], and S1p1 agonist administration was found to lead to collateral growth 7 days after unilateral CCAo [37]. One recent study highlighted the importance of EphA4 and the Angpt/Tie2 axis as targets for developing the collateral blood supply (remodeling, patency, and growth of pial vessels) after stroke, by vessel painting and laser speckle contrast imaging. Indeed, the pharmacological inhibition of EphA4 resulted in an increase in pial collateral size 4 days after pMCAo in CD1 mouse brain [42].

Ischemic preconditioning and/or postconditioning involves serial mechanical interruptions of BF and release (3 to 5 cycles), either in a cerebral artery or remotely in a lower limb. In rats aged 16–18 months, remote ischemic preconditioning (3 cycles of bilateral femoral BF occlusion/release) induced an early increase in collateral flow 4.5 h later [13]. Remote limb ischemic preconditioning (4 cycles of occlusion/release) in the mouse induces the enlargement of leptomeningeal anastomoses (latex perfusion) at the end of tMCAo [38]. Again, preconditioning was found to increase collateral anastomoses (measured by two-photon and LSC imaging) from 45 to 180 min after pMCAo in the rat [44]. Remote immediate postconditioning induces an increase in the diameter of leptomeningeal anastomoses (latex perfusion) 24 h after three-vessel occlusion in the mouse [39].

Interestingly, aerobic exercise has been shown to prevent the aging-related rarefaction of pial collaterals (after vessel painting) observed 3 days after pMCAo in rats [41]. In these studies, various imaging techniques and time points were used for evaluation, making it difficult to determine the precise timing of the establishment of the leptomeningeal collateral blood supply in the brains of mice and rats after stroke. Interestingly, whisker stimulation during the first 2 h after pMCAo has been shown to be insufficient to modify the collateral supply and stroke volume in C57BL/6 J and CD1 mice, contrary to findings for rats [40].

## Discussion

Regardless of whether stroke is due to pre- or post-Willis artery occlusion, the local drop in oxygen tension and levels of energetic substances and the resulting changes to the gradient pressure lead to downstream microvascular dilation and a decrease in local hemodynamic resistance throughout the entire ipsilateral hemisphere in cases of carotid artery occlusion, and locally in cases of post-Willis occlusion. In the case of post-Willis occlusion, the decrease in hemodynamic resistance imposes a rerouting of the BF into the leptomeningeal arterial anastomoses in the watershed zone of the vascular beds of the different cerebral arteries involved. In the case of pre-Willis carotid artery occlusion, rerouting into the Willis circle is established side-to-side, via the azygos artery and both the PcomAs in rodents, allowing reperfusion of the ACA and PCA territories, the vascular bed of the MCA being perfused via its borders by the leptomeningeal collaterals extending from the ACA and/or PCA. The proximal MCA can also be reperfused directly by the ACA and the PCA via the circle of Willis. This hemodynamic adaptation can account for the location of infarct lesions essentially in the MCA territory. In humans, the internal carotid artery gives rise to the ACA and MCA; PCA is a terminal branch of the vertebral-basilar arterial system. The ACA territory is relatively well protected in the presence of a patent anterior communicating artery, whereas the PCA can supply collateral BF through the cortical anastomoses [3].

Together, these data suggest that the recruitment of leptomeningeal collaterals — the secondary collateral pathways — requires at least a treatment capable of activating the vascular wall (by acting on the endothelial cells to increase vasodilation). Conversely, the presence of comorbid conditions (hyperglycemia, chronic hypertension) impairs the dynamic recruitment of collaterals [45].

It would be interesting to measure arterial blood pressure and to determine its relationship to collateral recruitment, but this would require a craniotomy and deep anesthesia, which would not be compatible with blood flow measurements, particularly in small rodents.

The time course of artery occlusion and re-flow is also a very important issue with respect to the presence, absence or extension of ischemic brain lesions. Acute artery occlusion induces leptomeningeal collateral BF within an hour of artery occlusion in individuals with preserved endothelial function. Progressive artery subocclusion triggers the arterial remodeling processes of the arterial segments of the circle of Willis over a period of weeks in rodents, or months in humans, with complementation by the cortical collateral blood supply system [8, 51]. The amplitude of collateral BF changes in a dynamic manner. In preclinical studies, the time to establishment of the collateral supply and its magnitude also depend on the persistence of the arterial occlusion and its duration, and endothelial integrity. In a recent study using a non-a priori metabolomics method in patients referred for acute ischemic stroke, S1P was investigated as a candidate biomarker, with plasma S1P determinations by ELISA within 7 days of symptom onset. The cortical collateral blood supply was assessed by calculating the Tan score based on computed tomographic angiography results. S1P level was found to be a promising predictor of the early establishment of cerebral collateral blood supply [52].

**Fig. 6:**
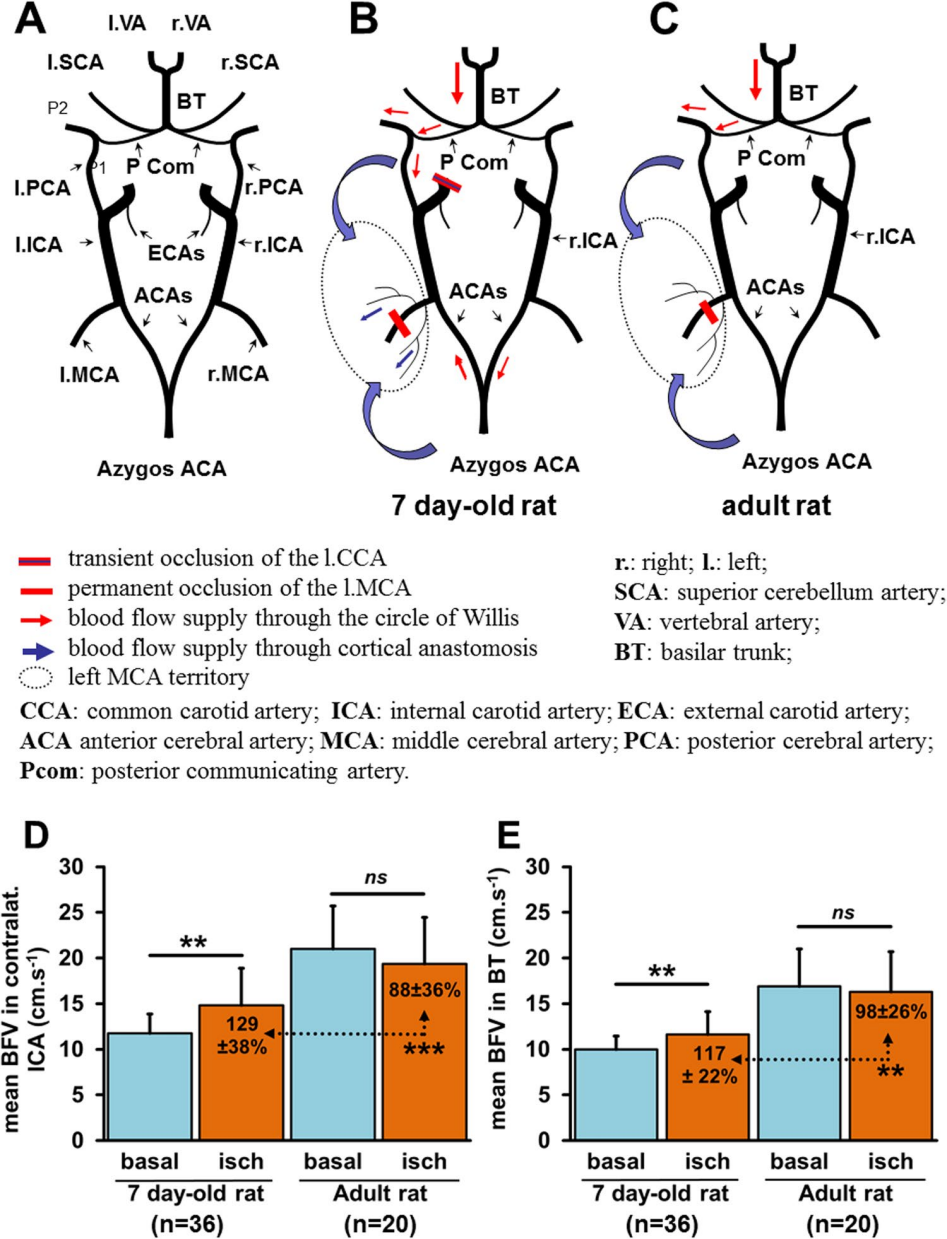
Collateral blood supply following focal ischemia in neonatal and adult rats. **A** Anatomy of the circle of Willis.** B** Permanent MCA occlusion (pMCAo) and transient CCA occlusion (tCCAo) are required to create an infarct in rat pups. **C** pMCAo is sufficient to create an infarct in adult rats. **D**, **E** One hour after focal ischemia, the increase in mean blood flow velocities in the contralateral ICA and BT provides evidence of the early establishment of the collateral blood supply through the circle of Willis and the cortical anastomoses in rat pups. No increase was observed in the adult rats at this time point. Data provided for neonatal stroke from references [9, 16, 32, 33], and for adult stroke from [55]

In healthy controls, PJ34 has been shown to recruit the arteriolar cerebral vasodilation reserve more strongly in neonatal mice than in adult mice, thereby playing a beneficial role during stroke [53]. Collateral BF may be established either during arterial occlusion or after reperfusion, depending on the age of the animal, and with the recruitment of one or both routes, depending on the experimental procedure. In 7-day-old rat pups, the collateral blood supply tends to be established very rapidly, immediately after the end of the ischemia procedure (50 min) when compared to most of adult rat models, in which the collateral blood supply is effective 2 to 3 h after the beginning of the procedure (Fig. 6). Aging may lead to the rarefaction of cerebral collaterals, leading to collateral recruitment failure with significantly impaired pial collateral dynamics (smaller diameter, decrease in red blood cell velocity), thereby aggravating ischemic injury by reducing penumbral blood flow [54]. Together, these data suggest that the collateral blood supply is stronger and established more rapidly in neonatal pups than in adults [8].

Villapol et al. showed that tCCAo (50 min) was insufficient to provoke an ipsilateral stroke in P7 rats, but nevertheless triggered apoptotic processes in the ipsilateral and contralateral hemispheres. At the end of ischemia, blood flow velocities (BFVels) in the contralateral ICA were about 50% higher, facilitating an increase in BFVels in the contralateral ACA followed by a reversal of BF in the ipsilateral ACA towards the ipsilateral MCA and ICA (reverse flow) and in the PCA. BFVels returned to basal levels 24 h after reperfusion [16]. These data suggest that a significant increase in blood flow in the contralateral hemisphere may activate transient molecular and cellular signaling pathways initiating aborted apoptosis. This effect was of short duration (48 h), disappearing when blood flow returned to normal levels. Apoptosis, for example, may result from an increase in O_2_ and NO levels at the time of reperfusion, due to both endothelial and neuronal NOS activity, and the formation of peroxynitrites and reactive oxygen species, which damage brain tissues [24, 56]. By contrast, in animals without reperfusion, the contralateral ICA first displays an increase in BF compensating for the persistent occlusion of the ipsilateral CCA, mirroring the efficiency of collateral BF via the communicating arteries of the circle of Willis, and contributing to the rapid opening of cortical anastomoses between the ACA and MCA territories. A dramatic increase in BF (a doubling of basal values) occurs in the contralateral ICA 24 h later, inducing cell death and illustrating the ongoing processes governing the establishment of collateral BF [16], without BF variation in the BT.

The anatomy of the circle of Willis in rodents remains incompletely known. According to most authors, the internal carotid artery (ICA) crosses the base of the skull and then gives rise to the three cerebral arteries, and the composition of the circle of Willis is as described above [15]. However, some authors rename the artery arising from the basilar trunk and leading to the distal segment of the PCA as the proximal PCA, whereas the proximal segment of the PCA is renamed the PcomA, as described by Lee on the basis of anthropomorphic analogies [57]. However, there are hemodynamic and morphological arguments against this view: (1) the proximal and distal segments of the PCA present BF in the same direction, (2) and have similar diameters, and (3) BFs forward from the ICA to the second part of the PCA. Conversely, the PcomA, arising from the basilar trunk and extending to the PCA, is very thin, is not always visible on color Doppler ultrasound acquisition, and presents an angle of about 60° with the distal segment of the PCA. In the case of ICA occlusion, collateral BF is established from the basilar trunk to the ICA through the ipsilateral PcomA; BF is reversed in the proximal segment of the PCA, being redirected towards the ICA, but the blood continues to flow forward in the distal segment of the PCA.

Physiologic imaging of the penumbra at the early stage in stroke patients represents a clinical target to precisely evaluate the proportion of brain tissue that can be salvaged by intravenous thrombolysis or mechanical arterial recanalization and/or recruitment of the leptomeningeal collateral circulation. It justifies the prescription of pharmacological adjuvant agents able to stimulate collateral circulation or neuroprotective treatment, particularly in case of arterial recanalization failure or contraindication. Ischemic stroke leads to localized cerebral hypo-perfusion that initiates a series of successive events that evolve over time: (1) excitotoxicity [58], (2) peri-infarct depolarization related to ATP deprivation and loss of ion channel activity [59], (3) nitrogen and oxygen free radical production leading to oxidative stress [60–62], (4) microvascular injury with blood–brain barrier disruption [63], (5) apoptosis [64], and (6) post-ischemic inflammation. Cerebral BF thresholds have been determined to underlie these events at the early phase of stroke: electrical failure occurs below a 40% drop in cerebral BF (gray matter: 50–55 mL/100 g/min), membrane failure, and tissues necrotize resulting in the extension of the core of lesion over the penumbra occur below a 20% drop [65, 66]. In case of early reinforcement of the collateral supply mediated by an efficient endothelial function within the minutes to few hours after artery occlusion, the blood supply may be sufficient to avoid a deep cellular hypoxia and the penumbra can then be salvaged counteracting the extension of the core of lesion [67]. Computed tomography (CT), magnetic resonance imaging (MRI), and positron emission tomography (PET) altogether give access to hemodynamic, metabolic, and cellular parameters that vary with the defined CBF thresholds as the cerebral blood volume (CBV), cerebral BF (CBF), cerebral metabolic rate of oxygen (CMRO_2_), oxygen extraction fraction (OEF), pH, lactate, and ATP tissue levels. These parameters can help to differentiate viable tissue at risk of progressing to infarction that can be rescued depending of early reinforcement of the collateral supply from tissue that is already infarcted, thus the inner and outer boundaries of ischemic penumbra. Cortical collateral circulation has been scored with CT angiography that allows for temporal discrimination between patients with poor from those with good collateral recruitment [68]. In addition, preclinical studies are needed to screen the pharmacological candidate molecules able to rescue the penumbra. Then, future trials including fast physiologic imaging examinations must be initiated to assess the effects of new treatments acting on the cortical collateral circulation or on brain neuroprotection that can rescue the penumbra in humans (see reviews of Leigh et al. 2018 [66], of Catanese et al. 2017 [68], and of Cheung et al. 2021 [69]).

In conclusion, as a means of limiting the size of the core of the lesion and its expansion over the penumbra, it is essential to promote the rapid establishment of arterial collateral BF following a cerebral artery occlusion. Both collateral systems can play a role in this process.

Collateral blood flow through the circle of Willis depends first and foremost on the anatomical integrity of this structure, including the presence of its different segments, such as the anterior and posterior communicating arteries in particular. When the circle is complete, the rerouting of the blood follows the changes in hemodynamic pressures induced by arterial occlusion, i.e., the resulting hemodynamic pressure gradients. Nevertheless, during the acute phase of the stroke, increasing systemic blood pressure entails a risk of hemorrhagic transformation or expansion of the ischemic area.

The cortical collateral blood supply depends on the integrity of the endothelial function stimulated downstream from the artery occlusion. Rapid progress in non-invasive neuroimaging and computational techniques (2D color-coded ultrasound imaging; MR angiography, CT angiography) has made it possible to evaluate changes in blood flow occurring during stroke in preclinical models and in clinical settings, and guidelines for the evaluation of collateral blood flow in stroke patients have recently been proposed [70]. Even if the recruitment of leptomeningeal collaterals appears to be unable to compensate completely for the impaired BF in patients with ICA and/or MCA steno-occlusive arterial lesions [71], the use of pharmacological agents capable of enhancing the collateral supply by stimulating endothelial function may be a promising therapeutic strategy. Many issues remain: which molecules best stimulate endothelial function and for how long? What is the most appropriate time window for treatment? How is efficacy affected by vascular risk factors? Which model best mimics human disease as a function of age? Which readouts should be used? The time course of collateral recruitment for the primary and/or secondary collateral pathways could be evaluated separately for transient and permanent arterial occlusion in rat and mouse brains, and at different developmental stages, to determine the best treatment for each collateral supply pathway and to ensure the best outcomes (cellular and/or behavioral). Caution is required in the direct extrapolation of these preclinical findings to humans, and clinical trials should be conducted to assess the added value of pharmacological drugs for stimulating collateral blood flow at appropriate time points.
